# Resident Cardiac Immune Cells and Expression of the Ectonucleotidase Enzymes CD39 and CD73 after Ischemic Injury

**DOI:** 10.1371/journal.pone.0034730

**Published:** 2012-04-13

**Authors:** Florian Bönner, Nadine Borg, Sandra Burghoff, Jürgen Schrader

**Affiliations:** Department of Molecular Cardiology, Heinrich-Heine-University Düsseldorf, Düsseldorf, Germany; University of Colorado Denver, United States of America

## Abstract

**Background:**

The ectoenzymes CD39 and CD73 are expressed by a broad range of immune cells and promote the extracellular degradation of nucleotides to anti-inflammatory adenosine. This study explored the abundance of CD73 and CD39 on circulating and resident cardiac leukocytes and coronary endothelial cells under control conditions and in response to inflammation following myocardial ischemia and reperfusion (I/R).

**Methods and Results:**

A method was elaborated to permit FACS analysis of non-myocardial cells (resident leukocytes, coronary endothelium and CD31^−^ CD45^−^ cells) of the unstressed heart. Under control conditions the murine heart contained 2.3×10^3^ resident leukocytes/mg tissue, the most prominent fraction being antigen-presenting mononuclear cells (CD11b^+^ CD11c^+^ F4/80^+^ MHCII^+^) followed by B-cells, monocytes and T-cells. CD73 was highly expressed on circulating and resident cardiac lymphoid cells with little expression on myeloid cells, while the opposite was true for CD39. Cardiomyocytes and erythrocytes do not measurably express CD39/CD73 and CD39 dominates on coronary endothelium. Three days after I/R, CD73 was significantly upregulated on invading granulocytes (2.8-fold) and T-cells (1.5-fold). Compared with coronary endothelial cells, CD73 associated with leukocytes comprised 2/3 of the total cardiac CD73.

**Conclusion:**

Our study suggests that extracellular ATP formed during I/R is preferentially degraded by CD39 present on myeloid cells, while the formation of immunosuppressive adenosine is mainly catalysed by CD73 present on granulocytes and lymphoid cells. Upregulated CD73 on granulocytes and T-cells infiltrating the injured heart is consistent with the existence of an autocrine adenosinergic loop which may promote the healing process.

## Introduction

Myocardial infarction leads to a sterile inflammatory response which aims to clear myocardial tissue from cell debris and to replace the destroyed cardiomyocytes by scar tissue in the process of cardiac wound healing [1]. This immune response is dependent on specific temporal and local activation of immune components [2]. Necrotic cells release damage associated molecular patterns (DAMPs) and stimulate the innate immune system (i.e. complement or toll-like receptors) [3]. DAMPs ingested by tissue macrophages can lead to the production of IL-1β and subsequently to the release of chemokines (i.e. MIP-2) which recruit granulocytes and inflammatory monocytes from the circulation and spleenic reservoirs [1], [4]. The clearance of dead cells and extracellular matrix (ECM) debris by innate immune cells after transendothelial migration is a key feature in this first phase of cardiac repair. Infiltration of granulocytes and monocytes peak at day 3 after ischemia/reperfusion (I/R) [1]. The inflammatory phase is followed by proliferation and ECM maturation in the course of myocardial healing [5]. Proper resolution of inflammation and transition into tissue remodeling is a prerequisite for cardiac healing [2]. Whether the unstressed heart contains resident immune cells, as has been described for the aorta [6], brain [7], skin [8], liver [9], and kidney [10], is not known.

It is becoming increasingly apparent that CD73-derived adenosine plays a key role in the regulation of inflammatory reactions by modulating endothelial adhesion, transmigration, T-cell activation and disease progression [11], [12]. Adenosine has been shown to act as a potent anti-inflammatory autacoid [3], and extracellular adenosine formation is generally thought to result from the sequential dephosphorylation of extracellular ATP to AMP by action of an ectonucleoside triphosphate diphosphohydrolase (CD39) followed by degradation to adenosine by ecto-5′-nucleotidase (CD73) [13]. Necrotic cells in myocardial infarction release ATP and cellular ATP release has also been reported for activated granulocytes and T-cells [14], [15]. The mechanism of nucleotide release appears to be cell-type specific and may involve membrane ion channels, ABC-transporters, and exocytotic granule secretion [16]. Also activation of the P2X7-receptor, present on immune cells, triggers ATP release [17]. While ATP primarily acts as a proinflammatory signal on purinergic P2 receptors, its degradation product adenosine signals through P1 purinergic receptors mediating both anti- and proinflammatory effects depending on the receptor subtype [18]. Since the affinity of these receptor subtypes for adenosine differs, the adenosine signalling largely depends on the interstitial adenosine concentration which is importantly modulated by abundance and activity of CD73 [19]. Generally, the abundance of the ectonucleotide cascade involving CD39 and CD73 determines whether P2 or which subtype of P1 receptors are preferentially activated and therefore if pro- or anti-inflammatory reactions are promoted [20].

While CD39 and CD73 have been described on numerous cell types including endothelial cells and immune cells [13], a detailed description of the expression of both enzymes on circulating and cardiac immune cells after I/R is lacking. Our study therefore explored the abundance of CD39 and CD73 on circulating and cardiac immune cells to obtain a first comprehensive overview on the dynamics of extracellular adenine nucleotide degradation. Furthermore, a method was optimized which enabled for the first time the reliable assessment of resident cardiac immune cells in the unstressed heart which formed the baseline for ischemia-introduced changes. Finally, enzyme expression on immune cells was compared with those on the coronary endothelium, platelets, and erythrocytes suggesting compartmentation of ATP degradation at the cellular level.

## Materials and Methods

### Mice

Animal experiments were performed in accordance with the national guidelines on animal care and were approved by the Bezirksregierung Duesseldorf. The female mice (C57BL/6, 18–23 g body weight, 8–12 weeks of age) used in this study were bred at the Tierversuchsanlage of Heinrich-Heine-University, Duesseldorf, Germany. They were fed with a standard chow diet and received tap water *ad libitum*.

### Cardiac Ischemia/Reperfusion

Myocardial infarction was provoked by ligation of the left anterior descending coronary artery (LAD). Mice were intubated and anesthetized by mechanical ventilation with isoflurane (1.5%) at a rate of 150 strokes/min and a body weight adapted tidal volume ranging between 225–275 µl. Each animal was placed in a supine position, the chest wall was shaved, and electrocardiogram (ECG) needles were inserted subcutaneously representing lead II to measure ST-segment elevations during myocardial infarction. All operations were performed under an upright dissecting microscope (Leica MS05). The chest was opened with a lateral cut along the left side of the sternum. Subsequently, the pericardium was gently dissected to allow visualization of coronary artery anatomy. Ligation was done with an 8-0 polypropylene suture with a tapered needle passed underneath the LAD, 2 mm from the tip of the left auricle. The LAD suture was threaded through a small piece of plastic tube (PE-10 tubing) with blunt edges, and two small weights (∼1 g) were attached to each end. With the weights freely hanging, the LAD was immediately occluded. The success of infarction was verified microscopically by paling of the myocardium as well as significant elevations of the ST-segment. LAD occlusion was terminated at once after 50 min, when the weights were removed. Successful reperfusion was assured by the reddening of the previous pale myocardium and termination of ST-elevation. The chest was then closed in layers with 6-0 polypropylene suture and Betaisodona was used for wound disinfection.

### Tissue digestion and cell isolation

The heart was rapidly removed from the thorax and perfused according to Langendorff with an oxygenated medium (perfusion pressure of 80 mmHg, 37°C) for 5 minutes containing 4 mM NaHCO_3_, 10 mM HEPES, 30 mM 2,3-butanedion-monoxime, 11 mM Glucose, 0.3 mM EGTA, 6.6 mM NaCl, 0.22 mM KCl, and 0.1 mM MgCl_2_×6H_2_O. Controlled tissue digestion was performed by perfusion with the same buffer in addition containing collagenase II (1050 U/ml, BioChrom AG, Berlin, Germany) for 35 min at 37°C. After removing non-ventricular tissue, hearts were weighted and subsequently mechanically dissociated with a McIlwain tissue chopper (TC752). Tissue was then suspended in 2% bovine serum albumin (BSA) buffer and gently dissociated by sequent pipetting steps. The cell suspension was first meshed through a 100 µm BD Falcon cell strainer and centrifuged at 55×g to separate cardiomyocytes from non-cardiomyocytes. Supernatant containing the non-cardiomyocyte fraction was again passed through a 40 µm mesh Filter (BD Bioscience).

Blood was collected by retroorbital puncture after injection of 2500 U heparin. For isolation of blood leukocytes whole blood was treated with ACK buffer (0.15 M NH_4_Cl, 1 mM KHCO_3_, 0.1 mM EDTA, pH 7.3) to lyse erythrocytes; thereafter cells were resuspended in MACS buffer (PBS, 0.5% BSA, 5 mM EDTA, pH 7.4).

To determine the recovery of CD45^+^ cells by the isolation procedure, hearts were processed as described above. Myocyte pellets and supernatants were separated and cells were disrupted with a tissue homogenizer (FastPrep-24, MP Biomedical). RNA isolation was performed using the QIAcube (Qiagen) following standard procedures. First-strand cDNA synthesis and quantitative real-time PCR was performed using the SuperScript® III Platinum® SYBR® Green Two-Step qRT-PCR Kit (Invitrogen) as described in the manual. Primers had the following sequence: CD45 forward 5′-ATTTGGGGATTCCAGAAACG-3′ and reverse 5′-TCCATGGGGTTTAGATGCAG-3′. At the end of PCR cycles, dissociation curve analysis was performed to ascertain the amplification of a single PCR product. To be able to quantitate the results obtained, a standard curve was generated using 5×10^6^ leukocytes from peripheral blood.

### mAbs and flow cytometry

Cells were resuspended in MACS buffer, preincubated with FcR Blocking Reagent (Miltenyi Biotech) and stained with the following antibodies: anti- **CD45**-APC, 30-F11 (BD Bioscience), -**CD11b**-PE, M1/70 (BD Bioscience), -**CD8**a-APC-H7, 53-6.7 (BD Bioscience), -**CD25**-PE-Cy7, PC61 (BD Bioscience), -**Ly-6C**-FITC, AL-21 (BD Bioscience), **TER-119**-PE, TER119 (BD Bioscience), -**CD45**-PE, 30F11 (Miltenyi Biotech), -**CD11b**-APC, M1/70.15 (Miltenyi Biotech), -**CD3**-APC, 145-2C11 (Miltenyi Biotech), **CD49b**-APC, DX5 (Miltenyi Biotech), **NKp46**-APC, 29A1.4 (Miltenyi Biotech), -**CD39**-PE-Cy7, 24DMS1 (eBioscience), -**CD4**-PerCP-Cy5.5, RM4-5 (eBioscience), -**Ly-6G (Gr-1)**-PerCP-Cy5.5, RB6-8C5 (eBioscience), -**CD45R(B220)**-APC-eFluor780, RA3-6B2 (eBioscience), -**F4/80**-APC-eFluor780, BM8 (eBioscience), -**CD11c**-PE-Cy7, N418 (eBioscience), -**CD73**-FITC, 496406 (R&D Systems), -**CD39**-FITC, 495826 (R&D Systems), -**CD31**-APC, 390 (Biolegend), **FoxP3**-PE, MF23 (BD Bioscience), **Ly6c**-APC-Cy7, AL-21 (BD Bioscience), **MHCII**-FITC, M5/114.15.2 (Miltenyi Biotech).

To identify the individual subsets of the total pool of CD45^+^ immune cells we used a panel of antibodies against different cell-specific leukocyte markers: cytotoxic T-cells (CD3^+^, CD8^+^), T-helper cells (CD3^+^, CD4^+^), regulatory T-cells (CD3^+^, CD4^+^, CD25^+^, FoxP3^+^), B-cells (CD45R(B220)^+^), NK cells (CD49b(DX5)^+^, NKp46^+^), granulocytes (CD11b^+^, Ly6g^+^), monocytes (CD11b^+^, Ly6g^−^, CD11c^−^, Ly6c^low/high^) and myeloid antigen-presenting cells (APCs; CD11b^+^, Ly6g^−^, CD11c^+^, F4/80^−/+^, MHCII^−/+^). For intracellular staining of Foxp3, we used the Foxp3-Staining Buffer Set (eBioscience) according to the manufacturer's protocol.

After 5 min of incubation at room temperature cells were washed and resuspended in 200 µl MACS buffer for flow cytometry. Cell measurements were performed with a FACSCanto II flow cytometer (BD Bioscience). For analysis, the placement of gates was based on fluorescence minus one (FMO) controls. The minimum number of events used to define a cell population was 150. The analysis was always performed on individual hearts.

For quantification of antigen density, the Quantum™ Simply Cellular® kit (Bangs Laboratories) was used. In short, this kit comprises 5 bead population coated with goat anti-mouse IgG which have different antibody binding capacities (ABC). The beads were stained with the same antibodies as the immune cells. By plotting the known ABC of each bead population against the measured median fluorescence intensity of the used antibody a standard calibration curve was generated. The calibration plot was used to determine the number of antibodies bound per cell from the median fluorescence intensity of each sample. CD39 or CD73 antigen density was only calculated for positive gated cells (CD39^+^ or CD73^+^ cells).

To exclude that enzyme digestion might have altered CD73 and CD39 expression, we performed ex vivo enzyme treatment of immune cells and could not observe any change in antigen expression (data not shown).

### Statistics

Unless otherwise stated, data are presented as mean values ± standard deviation. Results were analysed by graphical representation and by the Mann Whitney U Test or the Student's *t*-test, using SigmaPlot 11.0 (Systat Software).

## Results

### Resident leukocytes present in the unstressed heart

**Figure 1:**
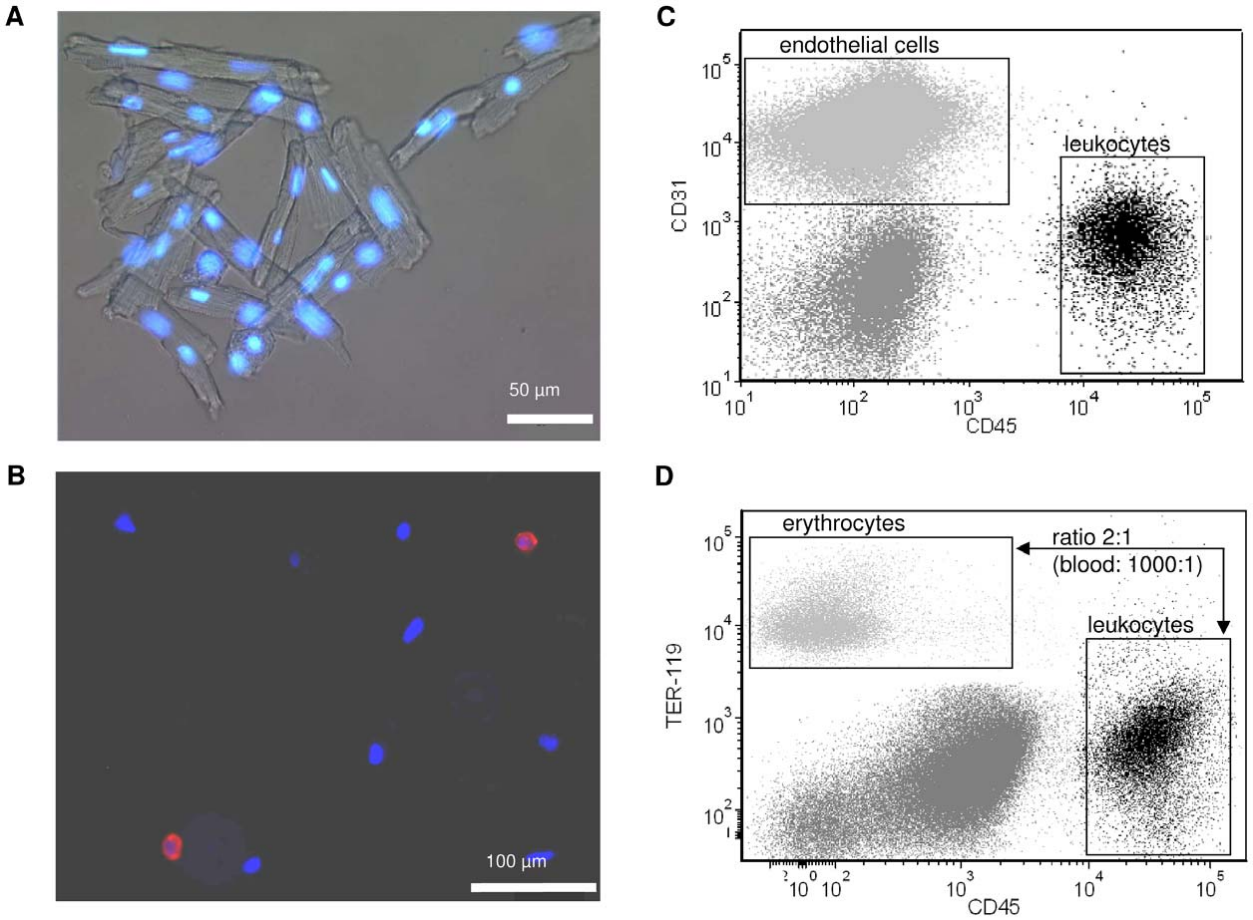
Analysis of the different cell fractions present within the unstressed heart. Fluorescence microscopy of intact murine cardiomyocytes (**A**) and non-cardiomyocytes (**B**) extracted from the murine heart. Red = CD45^+^ cells (fluorescence microscopy). Blue = nuclear stain with DAPI. **C**: Representative flow cytometry plot of non-cardiomyocytes. Black = CD45^+^ cells (lower right), light grey = endothelial cells (CD31^+^, upper left) and dark grey = CD31^−^ CD45^−^ (lower left). **D**: Representative Ter-119/CD45 plot of non-cardiomyocytes to derive the ratio of erythrocytes to leukocytes in myocardial tissue. Assuming a ratio of erythrocytes/leukocytes in peripheral blood to be 1000∶1, contamination of blood derived leukocytes was calculated to be <0.2% (n = 5).

To quantify resident cardiac immune cells, we elaborated and optimized the method for their reliable recovery from the unstressed heart. In brief, mouse hearts were perfused according to Langendorff with a calcium free medium containing collagenase and the myosin ATPase inhibitor 2,3-butanedione-monoxime, followed by mincing of tissue and sequential centrifugation steps as described in the *methods* section. The single cell suspension obtained by this procedure could conveniently be separated into fully intact rod-shaped cardiomyocytes and non-cardiomyocytes (Fig. 1A, 1B). Crucial for the analysis for non-cardiac cells is that cardiomyocytes remained intact to assure low background in FACS analysis. Further analysis of the non-cardiomyocyte fraction by flow cytometry yielded endothelial cells (CD31^+^), leukocytes (CD45^+^) and CD31^−^ CD45^−^ cells which comprise fibroblasts and smooth muscle cells (Fig. 1C). The endothelial cell fraction amounted to 20.0±9.1×10^3^ cells/mg heart tissue. Already the healthy, unstressed heart contained 2.27±0.91×10^3^ leukocytes/mg heart tissue (n = 5). To exclude a possible contamination with immune cells from circulating blood we determined the number of erythrocytes (TER-119^+^ cells) in cardiac tissue after perfusion and enzymatic digestion. Assuming the ratio of erythrocytes to leukocytes in peripheral blood to be 1000∶1, contamination of blood derived leukocytes was calculated to be <0.2% (n = 5) (Fig. 1D). In order to determine the recovery of leukocytes from cardiac tissue we quantified the mRNA of CD45 in the different fractions of the extraction process by RT-PCR and found that 77±4% (n = 4) of the entire cardiac mRNA of CD45 could be recovered by the cell isolation procedure.

**Figure 2:**
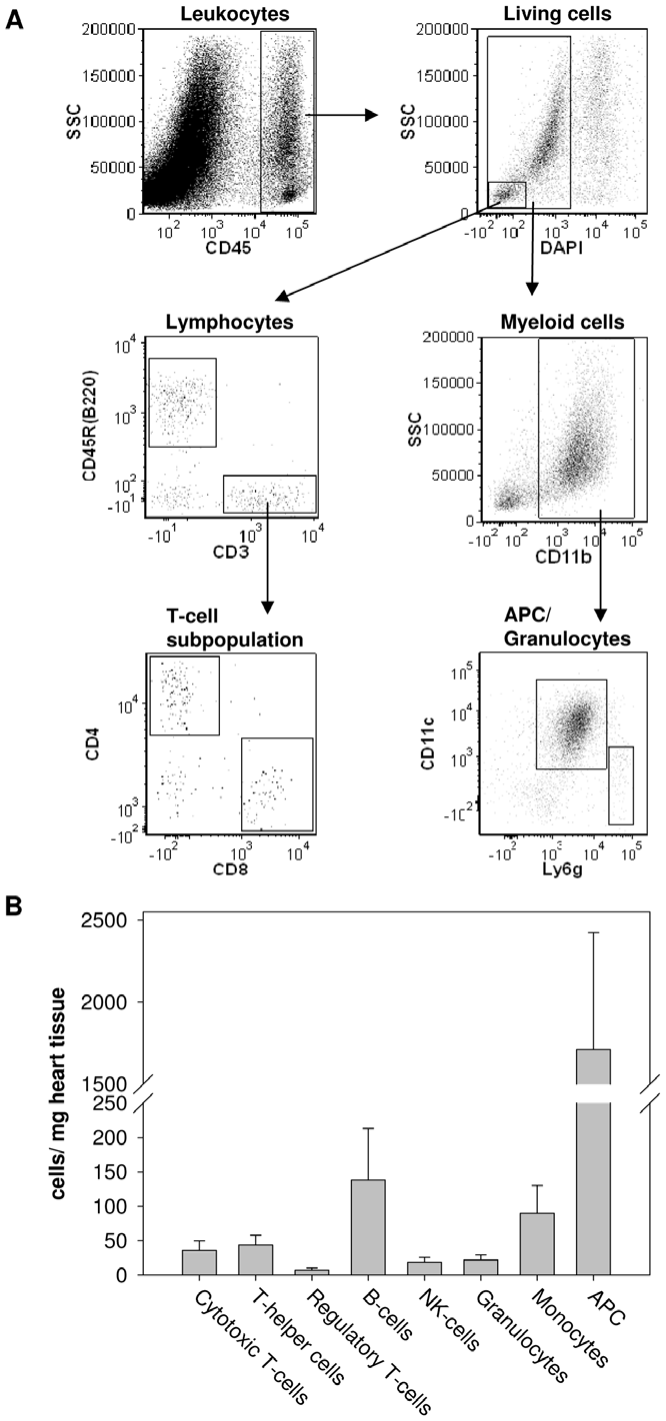
Leukocyte subpopulations in myocardial tissue under basal conditions. **A:** Gating strategy used to identify different leukocyte subpopulations by multiparametric flow cytometry. CD45^+^ cells were gated and DAPI-staining was used to exclude dead and apoptotic immune cells. Living CD45^+^ cells were then divided in subleukocyte populations using a panel of cell-specific fluorochrome-labeld antibodies. Lymphocytes were gated into CD45R(B220)^+^ cells (B-cells) and CD3^+^ cells (T-cells). T-cells were subdivided in CD4^+^ cells (T-helper cells) and CD8^+^ cells (cytotoxic T-cells). Myeloid cells were characterized as CD11b^+^ cells and further subdivided in CD11c^+^ cells (APCs) and Ly6g^+^ cells (granulocytes). **B:** Leukocyte subpopulations in the unstressed heart. Values are means ± SD of n = 5 experiments.

To further identify the individual subsets of CD45^+^ cells within the total non-cardiomyocyte cell pool, we used a panel of antibodies against different cell-specific leukocyte markers (see *methods*). The gating strategy shown in Figure 2 was used to identify different leukocyte subpopulations. As depicted in Figure 2B, the leukocyte population present within the heart mainly consisted of myeloid antigen-presenting cells (APCs = 73±2%; n = 5). APCs showed a common expression pattern for CD11b and CD11c, displayed high autofluorescence and 71±9% of APCs were additionally positive for the marker F4/80, 76±2% were positive for MHCII. T-cells, B-cells and monocytes were found in approximately equal numbers, comprising 87±30, 138±75, and 89±40 cells/mg heart tissue, respectively (Fig. 2B). Most of the monocytes were non-inflammatory CD11b^+^ Ly6c^low^ monocytes (75.3±16.7%, n = 5). The number of classical inflammatory cells like NK-cells and granulocytes were quite low comprising 18±7 and 22±8 cells/mg heart tissue, respectively.

### CD73 and CD39 expression on resident cardiac immune cells

**Figure 3:**
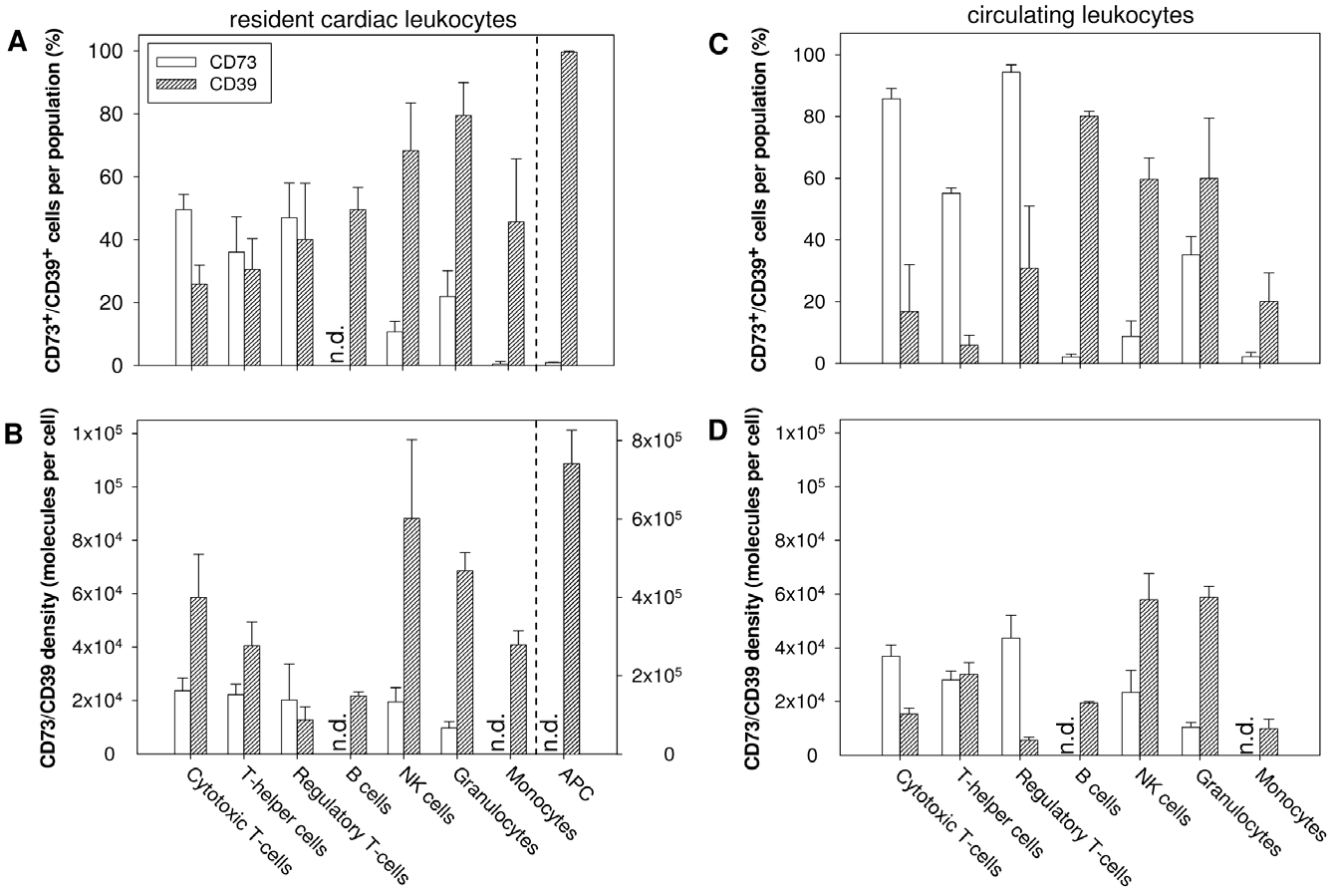
Abundance of CD73 and CD39 on leukocytes in blood and heart under basal conditions. **A**: CD73^+^ and CD39^+^ cells per leukocyte population in cardiac tissue. **B**: CD73/CD39 surface density on CD73^+^/CD39^+^ cells per leukocyte population in cardiac tissue. **C**: CD73^+^ and CD39^+^ cells per leukocyte population in blood. **D**: CD73/CD39 surface density on CD73^+^/CD39^+^ cells per leukocyte population in blood. Only positive gated CD73 or CD39 cells (Fig. 3A, 3C) were considered for the calculation of antigen density. Values are means ± SD of n = 5 experiments. n.d. = not detectable.

To estimate the contribution of the different cardiac immune cell populations to extracellular ATP degradation, we analysed the expression pattern of CD73 and CD39 on resident leukocytes in the heart (Fig. 3A, 3B) and on circulating leukocytes (Fig. 3C, 3D). As shown in Figure 3A, CD73 was mainly expressed on T-cells (>40%), whereas the expression on B-cells and myeloid cells was generally low except for granulocytes (22±8%). Figure 3B summarises data on CD73 density on the individual CD73^+^ leukocyte populations. As can be seen, the expression of CD73 per cell was similar in all T-cell populations and NK cells ranging between 20–24×10^3^ CD73 molecules per cell. The expression level on granulocytes was slightly lower with 9.77±2.41×10^3^ molecules per cell. Unlike CD73, CD39 could be detected on all immune cell populations. As shown in Figure 3A, the expression profile of CD39 is opposite to that of CD73: lowest expression was on T-cells and highest on myeloid cells. Interestingly, nearly all APCs expressed CD39, while CD73 was absent (Fig. 3A). The antigen density of CD39 was generally higher than that of CD73 except for regulatory T-cells. By far the highest expression level of CD39 was found with APCs reaching 741.8±84.7×10^3^ molecules per cell (Fig. 3B).

Since resident cardiac immune cells most likely originated from circulating blood, we analysed in a separate experimental series the expression of CD73 and CD39 on peripheral leukocytes (Fig. 3C, 3D). The general expression pattern of both ectoenzymes on leukocytes in the blood was generally similar to that in the heart (Fig. 3C). As can be seen, CD73 is mainly expressed on T-cells (∼55–94% cells within the different T-cell populations), whereas CD39 was preferentially expressed on B-cells (∼80%) and myeloid cells (∼59% of granulocytes and NK cells). Note, that the fraction of CD73^+^ T-cells and granulocytes was significantly lower in the heart compared to peripheral blood (e.g. cytotoxic T-cells: heart = 49.6±4.8%; blood = 85.7±3.3%, P<0.001; granulocytes: heart = 21.9±8%, blood = 35.1±6%, P = 0.02). In addition, when compared to blood the expression level of CD73 was significantly lower on cardiac cytotoxic T-cells (23.72±4.76×10^3^ versus 36.84±4.22×10^3^; P = 0.006) as well as on T-helper cells (22.22±3.93×10^3^ versus, 28.08±3.27×10^3^; P = 0.026). In contrast to CD73, the number of CD39^+^ leukocytes in myocardial tissue was generally higher compared to blood (Fig. 3A, 3C). The exception are B-cells exhibiting less CD39^+^ cardiac immune cells compared to peripheral blood (49.5±7.1% versus 80±1.6%; P = 0.008). Unlike CD73, antigen density of CD39 was higher on cardiac T-cells, granulocytes and monocytes compared to blood cells (Fig. 3B, 3D).

### Cardiac immune cells three days after I/R

**Figure 4:**
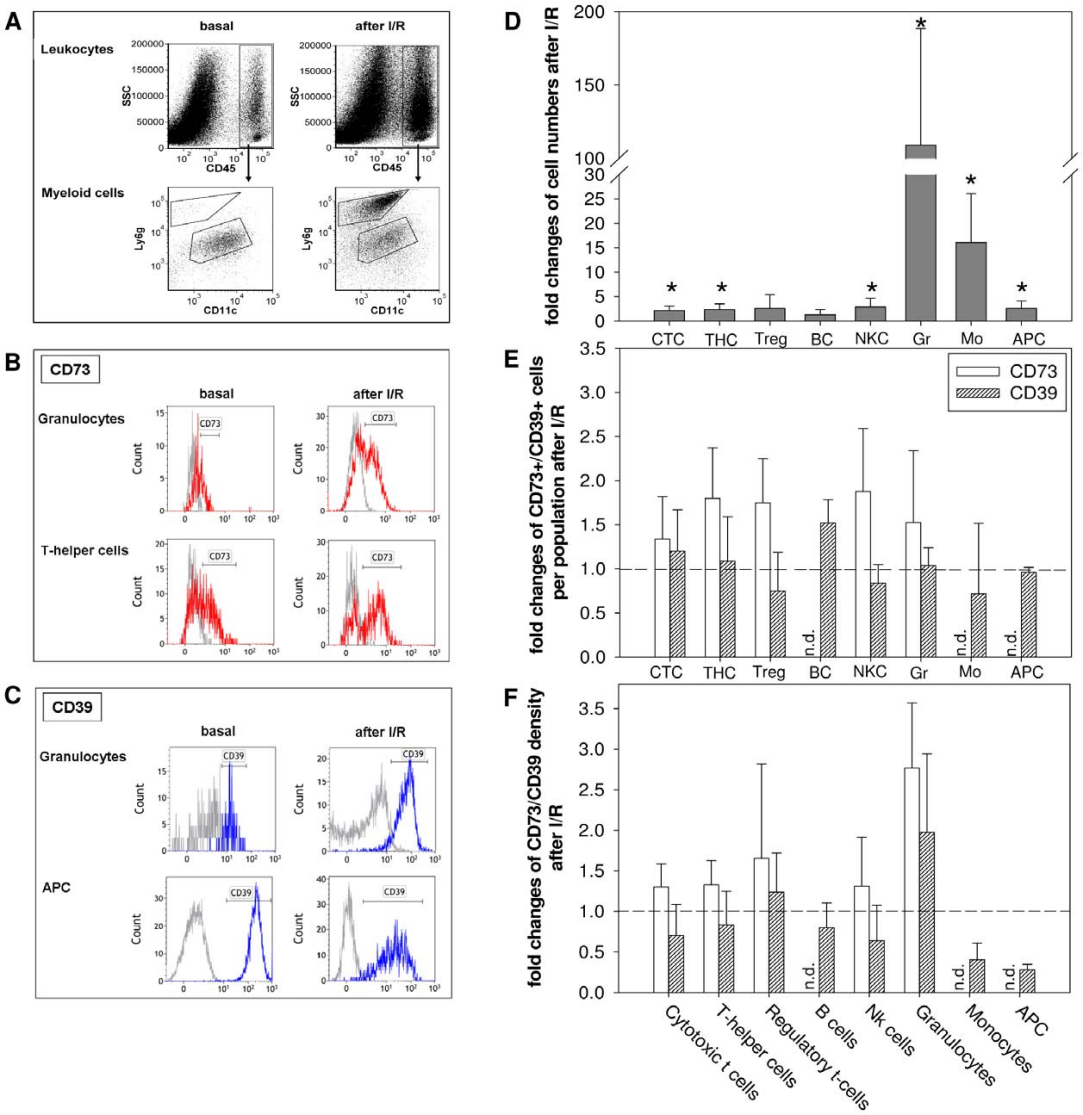
Changes of total leukocytes, CD73^+^/CD39^+^ cells and CD73/CD39 surface density after I/R. **A**: Representative flow cytometry plots of digested hearts under basal conditions compared to 3 days after I/R. **B**: Representative flow cytometry histograms of CD73 expression (red) under basal conditions compared to 3 days after I/R. Grey histograms = fluorescence minus one control (FMO). **C**: Representative flow cytometry histograms of CD39 expression (blue). Grey histograms = fluorescence minus one control (FMO). **D**: Increase of leukocyte populations within myocardial tissue after I/R. * P<0.05 for cells/mg heart tissue under basal conditions vs. I/R. CTC = cytotoxic t-cells, THC = T-helper cells, Treg = regulatory t-cells, BC = B-cells, NKC = NK cells, Gr = Granulocytes, Mo = Monocytes, APC = Antigen-presenting cells. **E**: Changes of CD73^+^/CD39^+^ cells per leukocyte population after I/R. **F**: Changes of CD73/CD39 surface density on CD73^+^/CD39^+^ leukocytes after I/R. Analysis was done 3 days after I/R. Values are means ± SD of n = 5 experiments. n.d. = not detectable.

When cardiac immune cell infiltration and expression of CD73/CD39 was analysed three days after I/R the total number of immune cells within the heart increased from 2.27±0.91×10^3^ to 9.11±3.34×10^3^ leukocytes/mg heart tissue (n = 5). As expected [21], this was mainly due to infiltrating granulocytes (∼100-fold increase) and monocytes (∼15-fold increase) (Fig. 4A, 4D). This increase of the monocyte population was mainly due to the infiltration of inflammatory CD11b^+^ Ly6c^high^ monocytes (83-fold increase) rather than non-inflammatory CD11b^+^ Ly6c^low^ monocytes (2.5-fold increase). Beside these phagocytic cells also cytotoxic T-cells (P = 0.005), T-helper cells (P = 0.014), NK cells (P = 0.017), and APCs (P = 0.008) were found to be significantly increased in the infarcted heart (Fig. 4D).

Figure 4E shows that the fraction of CD73^+^ T-cells, NK-cells and granulocytes in myocardial tissue increased 1.3–1.8 fold in the infarcted heart. B-cells, monocytes and APCs did not express CD73, similar to what was observed under basal conditions. In addition, we found the CD73 density to be increased (Fig. 4F). Notably, granulocytes expressed CD73 at significantly higher levels compared to basal conditions (2.8-fold increase; P<0.001, Fig. 4B). In contrast to CD73, the fraction of CD39^+^ cells remained largely unchanged when compared to the unstressed heart except that CD39^+^ B-cells significantly increased in the infarcted heart (1.5-fold; P<0.001, Fig. 4C) and CD39^+^ monocytes decreased (0.43-fold). Antigen density of CD39^+^ monocytes and APCs were found to be significantly decreased 0.4-fold (P = 0.004) and 0.3-fold (P<0.001) under I/R conditions (Fig. 4C).

**Figure 5:**
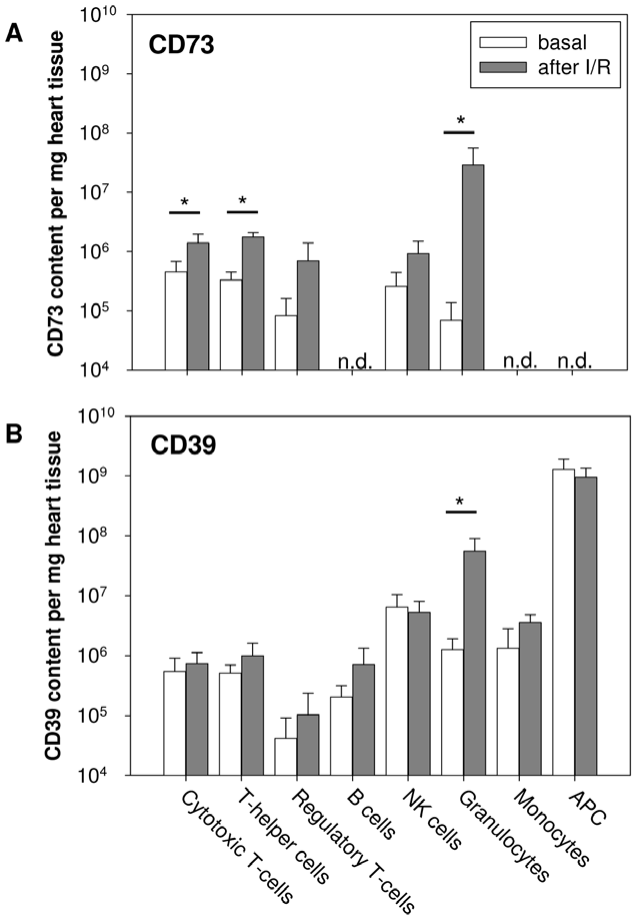
Calculated CD73/CD39 content per mg heart tissue under basal conditions and after I/R. The number of cell surface molecules was calculated by multiplying the absolute cell count/mg heart tissue with the percentage of CD73^+^/CD39^+^ cells and with the number of CD73/CD39 molecules on those cells. **A**: Calculated CD73 content per mg heart tissue. **B**: Calculated CD39 content per mg heart tissue. Values are means ± SD of n = 5 experiments. * P<0.05; n.d. = not detectable.

To investigate whether the changes in ectoenzyme expression after I/R were specific for leukocytes in heart tissue, blood immune cells were analyzed three days after I/R. We found that neither the percentage of CD73^+^ and CD39^+^ leukocytes nor the antigen density on the individual immune cell populations were altered (data not shown). Next we have calculated the total cardiac CD73/CD39 content by taking into account the leukocytes present within the entire heart, the percentage of CD39^+^/CD73^+^ leukocytes and their respective cell surface density. As shown in Figure 5A, CD73 content on leukocytes substantially increased after I/R, mainly as a result of infiltrating granulocytes (∼400-fold increase of CD73 content/mg tissue). The observed changes were mainly due to the high number of infiltrating granulocytes (Fig. 4D) and the upregulation of CD73 expression (Fig. 4F). CD39 content was found to be upregulated on granulocytes only (Fig. 5B).

### CD73 and CD39 expression on non-immune cells in the heart and blood

To obtain a comprehensive overview of the two nucleotide degrading ectoenzymes on immune and non-immune cells of mice, we have measured the expression of CD73 and CD39 on cardiomyocytes, coronary endothelial cells, and CD31^−^ CD45^−^ cells as well as on erythrocytes and platelets. From the data summarized in Table 1, it can be seen that cardiomyocytes and erythrocytes do not measurably express CD73 and CD39. While only a low fraction of coronary endothelial cells express CD73, these cells are highly positive for CD39. Interestingly, CD31^−^ CD45^−^ cells express no CD73, but a rather high fraction (65.1%) is positive for CD39. A similar distribution was found for platelets: they do not express CD73 but a sizable fraction is positive for CD39.

**Table 1:** Expression of CD73 and CD39 on cardiac cells and circulating blood cells under basal conditions (n = 4–5).

cell populations		CD73 (%)	CD39 (%)
cardiac cells	cardiomyocytes*	0	0
	coronary endothelial cells	1.9±0.3	99.4±0.3
	CD31^−^ CD45^−^ cells	0	65.1±12.9
blood	erythrocytes	0	0
	platelets	0	35.2±5.1

* Analysed by fluorescence microscopy.

**Figure 6:**
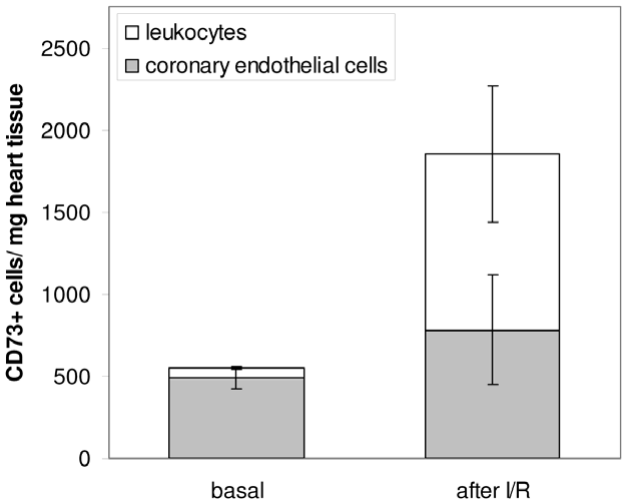
Comparison of cardiac CD73^+^ leukocytes and coronary endothelial cells under basal conditions and after I/R. Values are calculated from data given in Table 1. Values are means ± SEM of n = 5 experiments.

The values for CD73 reported above enabled us to compare total CD73 expression within the heart under control conditions and after I/R. As is shown in Figure 6, vascular endothelial cells from the unstressed heart accounted for the majority of CD73^+^ cells (90±2.6%; n = 5), and resident immune cells comprise only a very small fraction. However, three days after I/R the largest CD73^+^ cellular fraction consists of leukocytes (72.1±12.5%; n = 5) which have invaded the heart. Under these conditions we found the expression of CD39 on coronary endothelial cells to be downregulated by 18% (44.4±3.6×10^4^ versus 36.6±4.9×10^4^; P = 0.029).

## Discussion

Major findings of this study are that already the unstressed heart contains of about 2.3×10^3^ resident leukocytes/mg tissue, the most prominent fraction being myeloid APCs, which are likely to serve as sentinels of the myocardial immune system. The uneven distribution of CD39 and CD73 between myeloid and lymphoid cells in the heart suggests that ATP released in the course of I/R is first dephosphorylated by myeloid cells while immunosuppressive adenosine is preferentially generated by lymphoid cells. As a consequence of I/R the expression of CD73 was significantly increased on granulocytes and T-cells suggesting enhanced local formation of anti-inflammatory adenosine.

Collagenase digestion of the perfused heart combined with mechanical dissociation of the tissue, together with filtration and differential centrifugation steps, is often used for the isolation of intact ventricular myocytes [22], [23]. In the present study we have elaborated a tissue extraction procedure for non-cardiac cells and regularly recovered 77% of total leukocytes with negligible contamination from vascular blood cells. With the optimized procedure other non-cardiac cells such as coronary endothelial cells as well as CD31^−^ CD45^−^ cells comprising fibroblasts and smooth muscle cells can be equally well analysed by flow cytometry (Fig. 1). This for the first time permits the detailed analysis of resident immune cells in the unstressed heart. The procedure should be useful in future studies e.g. to study the role of APCs in immune defense, or to analyze the phenotype of coronary endothelial cells in the course of heart disease.

The largest fractions among resident immune cells within the unstressed heart are by far antigen-presenting cells. The most prominent APC cell fraction in the heart consists of CD11b^+^ CD11c^+^ F4/80^+^ MHCII^+^ cells. CD11c is wildly used as a classical marker for mouse dendritic cells (DC), whereas F4/80 generally is a macrophage marker. However, in the lung high levels of CD11c are also found on macrophages [24]. To clearly differentiate DC from macrophages in mice with conventional markers is known to be rather difficult particularly in non-lymphoid organs [25]. Aside of APCs there is a small fraction of resident B- and T-cells in the heart which is similar to resident immune cells in non-lymphatic organs such as liver [9], [26] and kidney [10]. Tissue-resident macrophages have been reported to protect liver from ischemia reperfusion injury via a heme oxygenase-1-dependent mechanism [9]. Interstitial dendritic cells form a contiguous network throughout the entire kidney and may form an immune surveillance network whose extent has not been fully appreciated yet [27]. The role of resident APCs in the heart is presently not known but it is likely that they, like in other organs, are activated by danger associated molecular patterns (DAMPs) after injury, secrete pro-inflammatory cytokines, activate T-cells and initiate neutrophil chemotaxis. APCs may therefore be important for cardiac protection (sentinel function) in response to injury as was already postulated for liver [9] and kidney [10].

The release of adenine nucleotides represents a critical first step for the initiation of purinergic signalling. Extracellular ATP can be derived from necrotic cells, but non-lytic ATP release has been reported for platelets [28], erythrocytes [29], and immune cells such neutrophils [14], monocytes/macrophages [30], and T-cells [15]. Once released, extracellular ATP can promote immune cell activation and pro-inflammatory responses by acting on P2 receptors [3]. For example, it was shown that ATP activates dendritic cells in lung [31] and skin [32] and is involved in the recruitment of phagocytotic cells [33]. The half-life of extracellular ATP is critically determined by the activity of CD39. The high activities of CD39 found on resident APCs and monocytes, on cardiac CD31^−^ CD45^−^ cells (fibroblasts, smooth muscle cells) and coronary endothelial cells suggest that various cardiac cells appear to synergize in the effective degradation of extracellular ATP to prevent ATP-induced cell death by activation of P2X_7_. By this mechanism extracellular ATP is kept low by abundantly expressed CD39 to terminate P2 receptor-mediated pro-inflammatory immune responses [34]. CD39 was also reported to be the dominant ectonucleotidase at the surface of mouse peritoneal and bone marrow-derived macrophages antagonizing the ATP-induced and P2X_7_ –mediated cell death [34],[35]. Similar to our findings, compartmentation of the both ectoenzymes was recently reported for lymph nodes in chronic lymphocytic leukemia patients [36]. In this study, CD39 was widely expressed while CD73 was restricted to proliferation centers suggesting that adenosine generation is locally confined.

CD39 and CD73 were found to be unevenly distributed among the different cardiac immune cells. Circulating and resident cardiac lymphoid cells highly expressed CD73 with little abundance on myeloid cells, while the opposite was true for CD39. In fact, resident cardiac APCs and monocytes showed no measurable CD73 but were highly positive for CD39. It thus appears that the initial step of ATP degradation in the heart is mediated almost exclusively by APCs, monocytes, and CD31^−^ CD45^−^ cells while the further dephosphorylation of AMP to adenosine occurs predominantly by lymphocytes and granulocytes. It should also be noted that the percentage of CD73^+^ immune cells is generally lower in the heart as compared to the circulating blood which might be either due to selective homing of CD73 negative immune cells or due to downregulation of CD73 when migrating through the endothelium into cardiac tissue. The latter hypothesis is supported by findings in a lymphocyte endothelial coculture model, in which CD73 activity was significantly decreased during adhesion and migration processes [37].

Consistent with data in the literature, we found after I/R profoundly increased numbers of granulocytes and monocytes within the heart [21]. As to the enzymes of the ectonucleotidase cascade, CD73 und CD39 were significantly upregulated on granulocytes under these conditions. While in the unstressed heart coronary endothelial cells contribute to 90% of the cell-associated CD73 in the heart (Fig. 6), this fraction dramatically changes after I/R when leukocyte-bound CD73 comprises about 2/3 of the entire CD73 within myocardial tissue. This difference is most likely even more pronounced when considering the local accumulation of immune cells at the site of inflammation. Similarly, we have observed a strong increase in CD73 expression on T-cells while CD39 remained unchanged. Consistent with this finding we recently reported upregulation of CD73 on Treg after antigenic stimulation which was associated with adenosine A2a receptor mediated downregulation of active NFkB and cytokine release [38]. In the context of cardiac function after I/R, the upregulation of CD73 on lymphocytes and granulocytes suggests an adenosinergic axis which becomes functionally relevant when necrosis and apoptosis lead to elevated extracellular nucleotide levels. When considering the local abundance of CD73, the accumulation of adenosine at the side of inflammation may be part of an autocrine signalling loop which limits the uncontrolled expansion of inflammation through activation of the A2a receptor. As already shown in other models, adenosine-mediated effects might include the regulation of neutrophil phagocytotic capacity [39] or inhibition of neutrophil transmigration into the tissue [40].

Similar considerations as for the myocardium may also be functionally relevant for the coronary vasculature. We found that endothelial CD39 after I/R was significantly downregulated which is similar to findings reported for kidney I/R [41], [42]. Complete lack of CD39 results in impaired endothelial barrier function [43] and disordered thromboregulation [44]. It therefore may be hypothesized that downregulation of endothelial CD39 in response to myocardial ischemia facilitates the infiltration of immune cells into the infarcted area.

In conclusion, the elaborated method of myocardial tissue dissociation enabled the reliable measurement of non-cardiac cells by flow cytometry in the unstressed heart. Among resident immune cells the most prominent fraction consisted of APCs acting most likely as sentinels for danger signals. Enzymes of the ectonucleotide cascade were unevenly distributed among the immune cells within the heart in that the initial degradation of extracellular ATP (CD39) is preferentially accomplished by myeloid cells while the further degradation of AMP to adenosine is catalysed by lymphoid cells. During myocardial I/R the upregulation of CD73 on infiltrating granulocytes favors the enhanced local formation of anti-inflammatory adenosine.

## References

[1] Rock KL, Latz E, Ontiveros F, Kono H (2010). The sterile inflammatory response.. Annu Rev Immunol.

[2] Frangogiannis NG (2008). The immune system and cardiac repair.. Pharmacol Res.

[3] Junger WG (2011). Immune cell regulation by autocrine purinergic signalling.. Nat Rev Immunol.

[4] Nahrendorf M, Pittet MJ, Swirski FK (2010). Monocytes: protagonists of infarct inflammation and repair after myocardial infarction.. Circulation.

[5] van den Borne SW, Diez J, Blankesteijn WM, Verjans J, Hofstra L (2010). Myocardial remodeling after infarction: the role of myofibroblasts.. Nat Rev Cardiol.

[6] Galkina E, Kadl A, Sanders J, Varughese D, Sarembock IJ (2006). Lymphocyte recruitment into the aortic wall before and during development of atherosclerosis is partially L-selectin dependent.. J Exp Med.

[7] Bulloch K, Miller MM, Gal-Toth J, Milner TA, Gottfried-Blackmore A (2008). CD11c/EYFP transgene illuminates a discrete network of dendritic cells within the embryonic, neonatal, adult, and injured mouse brain.. J Comp Neurol.

[8] Ochoa MT, Loncaric A, Krutzik SR, Becker TC, Modlin RL (2008). “Dermal dendritic cells" comprise two distinct populations: CD1+ dendritic cells and CD209+ macrophages.. J Invest Dermatol.

[9] Devey L, Ferenbach D, Mohr E, Sangster K, Bellamy CO (2009). Tissue-resident macrophages protect the liver from ischemia reperfusion injury via a heme oxygenase-1-dependent mechanism.. Mol Ther.

[10] Tadagavadi RK, Reeves WB (2010). Renal dendritic cells ameliorate nephrotoxic acute kidney injury.. J Am Soc Nephrol.

[11] Koszalka P, Ozuyaman B, Huo Y, Zernecke A, Flogel U (2004). Targeted disruption of cd73/ecto-5′-nucleotidase alters thromboregulation and augments vascular inflammatory response.. Circ Res.

[12] Eltzschig HK, Thompson LF, Karhausen J, Cotta RJ, Ibla JC (2004). Endogenous adenosine produced during hypoxia attenuates neutrophil accumulation: coordination by extracellular nucleotide metabolism.. Blood.

[13] Yegutkin GG (2008). Nucleotide- and nucleoside-converting ectoenzymes: Important modulators of purinergic signalling cascade.. Biochim Biophys Acta.

[14] Eltzschig HK, Eckle T, Mager A, Kuper N, Karcher C (2006). ATP release from activated neutrophils occurs via connexin 43 and modulates adenosine-dependent endothelial cell function.. Circ Res.

[15] Schenk U, Westendorf AM, Radaelli E, Casati A, Ferro M (2008). Purinergic control of T cell activation by ATP released through pannexin-1 hemichannels.. Sci Signal.

[16] Praetorius HA, Leipziger J (2009). ATP release from non-excitable cells.. Purinergic Signal.

[17] Pellegatti P, Falzoni S, Pinton P, Rizzuto R, Di VF (2005). A novel recombinant plasma membrane-targeted luciferase reveals a new pathway for ATP secretion.. Mol Biol Cell.

[18] Hasko G, Linden J, Cronstein B, Pacher P (2008). Adenosine receptors: therapeutic aspects for inflammatory and immune diseases.. Nat Rev Drug Discov.

[19] Fredholm BB, Chern Y, Franco R, Sitkovsky M (2007). Aspects of the general biology of adenosine A2A signaling.. Prog Neurobiol.

[20] Bours MJ, Swennen EL, Di VF, Cronstein BN, Dagnelie PC (2006). Adenosine 5′-triphosphate and adenosine as endogenous signaling molecules in immunity and inflammation.. Pharmacol Ther.

[21] Nahrendorf M, Swirski FK, Aikawa E, Stangenberg L, Wurdinger T (2007). The healing myocardium sequentially mobilizes two monocyte subsets with divergent and complementary functions.. J Exp Med.

[22] Fiset C, Clark RB, Larsen TS, Giles WR (1997). A rapidly activating sustained K+ current modulates repolarization and excitation-contraction coupling in adult mouse ventricle.. J Physiol.

[23] Zhou YY, Wang SQ, Zhu WZ, Chruscinski A, Kobilka BK (2000). Culture and adenoviral infection of adult mouse cardiac myocytes: methods for cellular genetic physiology.. Am J Physiol Heart Circ Physiol.

[24] Gonzalez-Juarrero M, Shim TS, Kipnis A, Junqueira-Kipnis AP, Orme IM (2003). Dynamics of macrophage cell populations during murine pulmonary tuberculosis.. J Immunol.

[25] Geissmann F, Gordon S, Hume DA, Mowat AM, Randolph GJ (2010). Unravelling mononuclear phagocyte heterogeneity.. Nat Rev Immunol.

[26] Novobrantseva TI, Majeau GR, Amatucci A, Kogan S, Brenner I (2005). Attenuated liver fibrosis in the absence of B cells.. J Clin Invest.

[27] Soos TJ, Sims TN, Barisoni L, Lin K, Littman DR (2006). CX3CR1+ interstitial dendritic cells form a contiguous network throughout the entire kidney.. Kidney Int.

[28] Gachet C (2006). Regulation of platelet functions by P2 receptors.. Annu Rev Pharmacol Toxicol.

[29] Arciero JC, Carlson BE, Secomb TW (2008). Theoretical model of metabolic blood flow regulation: roles of ATP release by red blood cells and conducted responses.. Am J Physiol Heart Circ Physiol.

[30] Wong CW, Christen T, Roth I, Chadjichristos CE, Derouette JP (2006). Connexin37 protects against atherosclerosis by regulating monocyte adhesion.. Nat Med.

[31] Idzko M, Hammad H, van Nimwegen M, Kool M, Willart MAM (2007). Extracellular ATP triggers and maintains asthmatic airway inflammation by activating dendritic cells.. Nature Medicine.

[32] Mizumoto N, Kumamoto T, Robson SC, Sevigny J, Matsue H (2002). CD39 is the dominant Langerhans cell associated ecto-NTPDase: Modulatory roles in inflammation and immune responsiveness.. Nature Medicine.

[33] Elliott MR, Chekeni FB, Trampont PC, Lazarowski ER, Kadl A (2009). Nucleotides released by apoptotic cells act as a find-me signal to promote phagocytic clearance.. Nature.

[34] Robson SC, Sevigny J, Zimmermann H (2006). The E-NTPDase family of ectonucleotidases: Structure function relationships and pathophysiological significance.. Purinergic Signal.

[35] Levesque SA, Kukulski F, Enjyoji K, Robson SC, Sevigny J (2010). NTPDase1 governs P2X(7)-dependent functions in murine macrophages.. European Journal of Immunology.

[36] Serra S, Horenstein AL, Vaisitti T, Brusa D, Rossi D (2011). CD73-generated extracellular adenosine in chronic lymphocytic leukemia creates local conditions counteracting drug-induced cell death.. Blood.

[37] Henttinen T, Jalkanen S, Yegutkin GG (2003). Adherent leukocytes prevent adenosine formation and impair endothelial barrier function by Ecto-5′-nucleotidase/CD73-dependent mechanism.. J Biol Chem.

[38] Romio M, Reinbeck B, Bongardt S, Huls S, Burghoff S (2011). Extracellular purine metabolism and signaling of CD73-derived adenosine in murine Treg and Teff cells.. Am J Physiol Cell Physiol.

[39] Salmon JE, Cronstein BN (1990). Fc gamma receptor-mediated functions in neutrophils are modulated by adenosine receptor occupancy. A1 receptors are stimulatory and A2 receptors are inhibitory.. J Immunol.

[40] Save S, Mohlin C, Vumma R, Persson K (2011). Activation of adenosine A2A receptors inhibits neutrophil transuroepithelial migration.. Infect Immun.

[41] Robson SC, Kaczmarek E, Siegel JB, Candinas D, Koziak K (1997). Loss of ATP diphosphohydrolase activity with endothelial cell activation.. Journal of Experimental Medicine.

[42] Candinas D, Koyamada N, Miyatake T, Siegel J, Hancock WW (1996). Loss of rat glomerular ATP diphosphohydrolase activity during reperfusion injury is associated with oxidative stress reactions.. Thrombosis and Haemostasis.

[43] Eltzschig HK, Ibla JC, Furuta GT, Leonard MO, Jacobson KA (2003). Coordinated adenine nucleotide phosphohydrolysis and nucleoside signaling in posthypoxic endothelium: role of ectonucleotidases and adenosine A2B receptors.. J Exp Med.

[44] Enjyoji K, Sevigny J, Lin Y, Frenette PS, Christie PD (1999). Targeted disruption of cd39/ATP diphosphohydrolase results in disordered hemostasis and thromboregulation.. Nat Med.

